# Are marginalized women being left behind? A population-based study of institutional deliveries in Karnataka, India

**DOI:** 10.1186/1471-2458-12-30

**Published:** 2012-01-12

**Authors:** Paul C Adamson, Karl Krupp, Bhavana Niranjankumar, Alexandra H Freeman, Mudassir Khan, Purnima Madhivanan

**Affiliations:** 1 School of Medicine, University of California, San Francisco, CA, USA; 2 Public Health Research Institute of India, Mysore, Karnataka, India; 3 School of Medicine, George Washington University, Washington, DC, USA; 4 Department of Community Medicine, Mysore Medical College & Research Institute, Mysore, KA, India; 5 Department of Epidemiology, Robert Stempel College of Public Health & Social Work, Florida International University, Miami, FL, USA

**Keywords:** Reproductive health, Millennium Development Goals, Maternal mortality, India, Women's health

## Abstract

**Background:**

While India has made significant progress in reducing maternal mortality, attaining further declines will require increased skilled birth attendance and institutional delivery among marginalized and difficult to reach populations.

**Methods:**

A population-based survey was carried out among 16 randomly selected rural villages in rural Mysore District in Karnataka, India between August and September 2008. All households in selected villages were enumerated and women with children 6 years of age or younger underwent an interviewer-administered questionnaire on antenatal care and institutional delivery.

**Results:**

Institutional deliveries in rural areas of Mysore District increased from 51% to 70% between 2002 and 2008. While increasing numbers of women were accessing antenatal care and delivering in hospitals, large disparities were found in uptake of these services among different castes. Mothers belonging to general castes were almost twice as likely to have an institutional birth as compared to scheduled castes and tribes. Mothers belonging to other backward caste or general castes had 1.8 times higher odds (95% CI: 1.21, 2.89) of having an institutional delivery as compared to scheduled castes and tribes. In multivariable analysis, which adjusted for inter- and intra-village variance, Below Poverty Line status, caste, and receiving antenatal care were all associated with institutional delivery.

**Conclusion:**

The results of the study suggest that while the Indian Government has made significant progress in increasing antenatal care and institutional deliveries among rural populations, further success in lowering maternal mortality will likely hinge on the success of NRHM programs focused on serving marginalized groups. Health interventions which target SC/ST may also have to address both perceived and actual stigma and discrimination, in addition to providing needed services. Strategies for overcoming these barriers may include sensitization of healthcare workers, targeted health education and outreach, and culturally appropriate community-level interventions. Addressing the needs of these communities will be critical to achieving Millennium Development Goal Five by 2015.

## Background

It has been more than a decade since the Millennium Development Goals (MDG) were adopted by the United Nations. Fueled by optimism that economic growth in the developing world would translate into reductions in health disparities, the MDGs were designed to focus attention on morbidity and mortality among some of the world's most vulnerable groups. MDG 5, for instance, targeted a two-thirds reduction in the death of mothers living in some of the poorest nations on the globe between 1990 and 2015. While this and other goals have clearly been helpful in mobilizing governments to make healthcare available to all, much remains to be done.

While many nations have made significant progress in reducing maternal death, improvements have been uneven both among countries and populations. Worldwide, more than a thousand women still die each day from pregnancy-related causes [1]. More than 99% of these deaths occur in the developing world; 87% in sub-Saharan Africa and South Asia-most in just 11 countries. Not surprisingly, given its huge population, India leads the world in total deaths with 63,000 mothers dying each year [2].

While annual maternal mortality in India appears staggering at first glance, the world's second most populous country has made significant progress toward attainment of MDG Five [3]. With a 4.9% rate of decline in the maternal mortality ratio (MMR) between 1990 and 2008, it is one of the few countries achieving reductions close to those required for success in 2015 [4]. Regionally, India's MMR of 230 per 100,000 population ranks better than Nepal's, Bangladesh's, Pakistan's and Myanmar's with MMRs of 380, 340, 260 and 240 respectively [2]. As in other parts of the world however, overall gains obscure differences between populations [5]. Estimates from the Government of India National Family Health Survey (NFHS II, 1998-1999) suggest for instance, that maternal mortality could be as much as 132% higher outside of cities [6]. In spite of these disparities most areas of India have still seen steady if not spectacular progress in reducing maternal deaths [7].

Much of India's current success in rural areas may be attributable to the establishment of the National Rural Health Mission (NRHM). The goal of NRHM was to provide accessible and affordable primary health care to the non-urban poor of India [2]. This was largely accomplished by revitalizing and staffing more than 22,000 Community Health Centres (CHCs), 4,000 Primary Health Centres (PHCs), and some 150,000 Sub-Centres (SCs) to serve India's rural populations [3]. Additionally, the government has hired almost a half million Accredited Social Health Activists (ASHAs) to promote health programs, and extended hours of operation for CHCs and PHCs [4]. While many still complain of glaring deficiencies in NRHM efforts [6], the clear progress being made in reducing maternal mortality in rural areas speaks loudly for the current health strategy.

Attaining further declines in MMR in India however, is likely to become increasingly difficult as more traditional interventions are exhausted. Some progress appears possible through additional investment in healthcare infrastructure for the rural poor. A recent survey found that nearly 150,000 health centers still do not have a doctor according to India's health ministry [8]. While activists continue to complain about unevenness of healthcare resources, the NRHM has made significant progress in staffing CHCs and PHCs although there remains a severe shortage of physicians and nurses trained in emergency obstetric care [9,10]. As more rural health centers achieve adequate staffing levels however, further reductions in MMR can only come from reaching harder-to-access populations, many of them residing in more isolated rural areas.

Beginning in 1935, the Government of India created a schedule of disadvantaged castes and tribes living in remote areas of the country for additional protection and services [11]. Labeled by higher Hindu castes as "untouchables" or "Dalits", these groups were often severely impoverished and faced widespread stigma and discrimination [12]. Scheduled Castes and Scheduled Tribes (SC/ST) had the shortest life expectancy at birth, the lowest rates of female literacy, and the highest infant and maternal mortality rates [11,13]. Even today, these SC/ST are among the most economically deprived and marginalized groups in India [14]. Most are rural, scattered, difficult to reach [15,16]. While they make up only 24% of India's total population [8], a 2008 study by UNICEF concluded that they contribute more than half of the country's maternal mortality [17]. Karnataka state, where the study was carried out, is one of the top ten states in India for SC/ST [18].

The data presented in this paper was collected in a population-based study of mothers in 16 randomly selected rural villages in Mysore *Taluk*, a subdivision of Mysore District, Karnataka state, between August and September, 2008. All households in each of the selected villages were fully enumerated and data from 1,342 households collected on demographics, caste, antenatal care, and institutional delivery from all mothers with children six or less years of age.

## Methods

### Study setting and sample selection

Mysore district in Karnataka had a population of 2.64 million in the 2001 census, with 63% of the population being rural [19]. The district consists of seven *Talukas *(sub-districts), of which Mysore *Taluk *is the most populated with 1.04 million. For the purpose of sampling, we listed all the villages in Mysore *Taluk *based on their population. There were 144 villages in Mysore *Taluk *and we then randomly selected 16 villages from this list and enumerated every household and resident. Household was defined as a group of people sharing one roof to live under and one kitchen. Resident was defined as person who has lived in that village for more than 6 months prior to the date of the survey.

### Ethical approval

The study was approved by the Independent Ethics Committees of Vikram Hospital and Public Health Research Institute of India, in Mysore.

### Data collection

Between August and September 2008, all households in sample villages were enumerated to determine eligibility. Households were eligible if they had any children 6 years of age or younger and the mother of those children expressed willingness to participate. If no one in the household could be questioned, or if residents of a household refused to participate in the survey, the house was marked as 'missing'. 'Missing' houses were factored into the calculation for overall response rate. Mothers in eligible households underwent a short structured cross-sectional survey on maternal healthcare.

Trained field staff obtained verbal consent from eligible residents to participate in the study. All eligible participants answered a short survey in *Kannada *(the local language) that obtained demographic data on mother including age, caste, possession of a below poverty line (BPL) card, and number of children 6 years of age or younger. In addition, interviewers collected information on the mother's most recent delivery including whether and where she had received antenatal care, what type of assistance she had at the time of delivery, and the location of delivery. Information on whether she had assistance during her delivery and the venue of each birth were collected for the most recent delivery.

### Data analysis

Data were analyzed using Stata 10.1 (Stata Corporation, College Station, TX). Descriptive statistics were used to provide a general profile of the study population. Since data were obtained through a survey administered to a random sample of the target population, all statistical analyses were conducted using the survey design command in Stata, which controlled for both inter- and intra- village variance. Pearson chi-squared or Fisher-exact tests were used for categorical variables and *t-tests *for comparison of continuous variables. Multivariable logistic regression analysis was used to estimate the association of variables with receiving antenatal care. Crude and adjusted odds ratios were calculated along with 95% confidence intervals.

## Results

There were a total of 3,830 households in the 16 randomly selected villages enumerated during the period of the study. Of these households, 3,616 (94.4%) agreed to participate in a screening interview, and 1,342 (35.0%) had at least one child 6 years of age or younger. Survey results are presented for mothers interviewed in the 1,342 eligible households.

### Demographics

Only one in four mothers identified themselves as being part of a general caste which is not designated as deprived (22%; 303/1342), 15.9% (213/1342) reported being part of a Scheduled Tribe (ST), 27.8% (373/1342) Scheduled Caste (SC), and 33.3% (447/1342) reported belonging to 'Other Backwards Castes' (OBC), another poverty category that is low-income but generally less impoverished than Scheduled Castes and Tribes. Six respondents (0.4%) refused to answer the question. A large majority of households (69.7%; 936/1342) said they possessed a 'Below Poverty Line' (BPL) card entitling them to 35 kg of food grain every month and data was missing for 15 respondents (1.1%). At the time of the survey, mothers were an average of 24.3 years (median: 23 years; range: 14-50 years) of age, while the average age of the mother at the time of her first delivery was 20 years (median: 19.5; range: 11-46 years).

Of the households surveyed, 55.1% (740/1342) had one child 6 years of age or younger, 41.0% (550/1342) had two children in that age category, 3.8% (51/1342) had three children, and one household (0.1%) had four children who were 6 years of age or younger. The median age of the most recently born child was 2 years (range: 0-6 years).

Almost all of the households (94.3%, 1266/1342) reported having at least one prenatal care visit during their most recent pregnancy. Almost all of those visits (92.1%; 1236/1342) were to a doctor, 1.8% (24/1342) to an Auxiliary Nurse Midwife (ANM), and 5.7% (76/1342) to some other type of healthcare provider. Data on antenatal care were missing for six households (0.4%).

### Delivery venue and assistance

**Figure 1:**
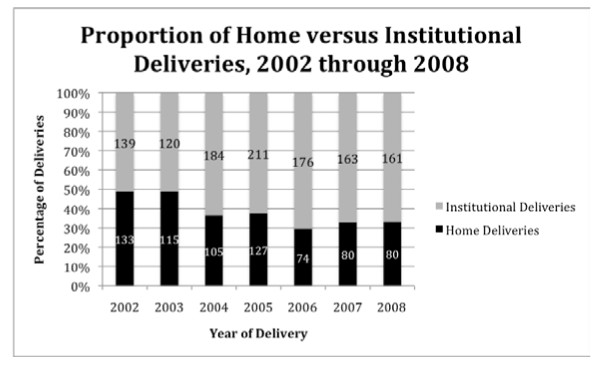
**Proportion of institutional deliveries versus home deliveries in Mysore *Taluk***.

For their most recent delivery, 55.4% (743/1342) of households reported delivering in the hospital, 1.3% (18/1342) at a Primary Health Center (PHC), 35.9% (482/1342) at home, and 7.4% (99/1342) either at another venue or data were missing. 'Institutional deliveries' were defined as deliveries taking place at a hospital or PHC. Among mothers surveyed, the percentage of institutional deliveries increased from 51% in 2002 to 70% in 2008 (Figure 1).

Approximately half (51.4%; 690/1342) of households received delivery assistance from a doctor, a small minority (9.4%; 126/1342) received delivery assistance from an ANM for their most recent delivery, while 26.1% (350/1342) utilized a Traditional Birth Attendant (TBA) and 7.6% (102/1342) a family member or neighbor. Few households (5.5%; 74/1342) did not provide responses on this topic.

#### Regression analysis

In a simple regression analysis, women who reported not receiving antenatal care had 6.4 (OR = 6.4; 95% CI: 3.72, 10.99) times higher odds than those who received antenatal care, to report a home delivery for their most recent child. Women who reported using a TBA for their most recent delivery had 3.6 times higher odds of not receiving antenatal care (OR = 3.6; 95% CI: 2.27, 5.80), than those reporting skilled assistance (doctor or ANM).

In multivariable analysis, which adjusted for inter- and intra-village variance, BPL status, caste, and receiving antenatal care were all associated with institutional delivery for the most recent child. Having received antenatal care was also associated with an eight-fold increase in odds (aOR = 7.95; 95% CI: 3.99, 15.82) of an institutional delivery after adjusting for village correlation, BPL status, and caste. Households not having a BPL card were at increased odds (aOR = 1.49; 95% CI: 1.15, 1.92) of reporting an institutional delivery compared with those who reported having a BPL card. Finally, households belonging to OBC or general castes were almost twice as likely to have an institutional delivery compared with those belonging to Scheduled Caste or Tribe (aOR = 1.87; 95% CI: 1.21, 2.89), after adjusting for village correlation, BPL status, and antenatal care (Table 1).

**Table 1:** Multivariable Logistic Regression Model with demographic and delivery characteristics and their associations with institutional deliveries among 1,342 rural households in Mysore *Taluk*, India

	Institutional Delivery			
**Characteristic**	**OR**^**1**^	**95% CI**	**aOR**^**2**^	**95% CI**

**Received Antenatal Care**				

No	Ref**	-	Ref	-

Yes	8.59*	4.02, 18.3	7.95*	3.99, 15.82

**Possess a "Below Poverty-Line" Card**				

No				

Yes	0.69*	0.52, 0.89	0.67*	0.52, 0.87

**Castes**				

General or 'Other Backwards Castes'	Ref	-	Ref	-

Scheduled Castes or Scheduled Tribes	0.47*	0.29, 0.76	0.54*	0.34, 0.83

^1^OR: Odds Ratio from univariate logistic regression

^2^aOR: Adjusted Odds Ratio from multivariable regression

* Significant at P-value ≤ 0.05

** Logistic regression assigned OR = 1.00 for the referent category for each variable

## Discussion

This population-based study found that institutional deliveries in rural areas of Mysore District had increased from 51% to 70% between the years of 2002 and 2008. Additionally, it showed that while significantly more mothers were seeking antenatal care and delivering in institutional settings, large disparities continued to exist in the uptake of maternal health services among different castes. Mothers belonging to OBC or general castes were almost twice as likely to have an institutional birth as compared to SC/ST.

The survey also found that a vast majority of women (93.4%) had received at least one antenatal checkup for their most recent pregnancy, a slightly higher percentage than the 88.6% reported for rural Karnataka in District Level Household Survey carried out in 2007/2008 (DLHS-3) [20]. In contrast, reported institutional deliveries were slightly lower (55.4%) compared to DLHS-3 (59.7%). These differences may reflect dissimilarities in demographic composition of the samples: DLHS-3 had only 18.1% SC/ST compared with 43.7% in our study.

Data from the survey also suggests that traditional birth attendants (TBA) still play an important role in deliveries in rural Karnataka. One out of every three births in sample villages occurred at home with the help of a traditional birth attendant. Not surprisingly, women who reported delivering at home were also significantly less likely to say they had received antenatal care. SC/ST reported both lower levels of antenatal care and poorer rates of institutional delivery.

Consistent with other studies, low income (as measured by possession of a BPL card) was associated with lower rates of institutional birth [21]. This finding suggests that simply making more maternity services available, particularly if they are for-profit in nature, is unlikely to solve the problem of low institutional delivery rates. Interestingly, our finding that SC/ST had lower odds of institutional delivery even after adjusting for possession of a BPL card, suggests that there might be even deeper social and cultural reasons for low uptake of services among these groups. Saroha et al., in their study of the relationship of caste and use of maternal health services, suggested that because maternal care involves physical contact with health service providers who often belong to general or other castes, lower caste women often elect home deliveries with TBA from their community out of fear of being stigmatized and discriminated against [16]. Such a finding may also suggest the need for targeted health promotion to increase the uptake of maternal health services. There is some evidence to suggest that SC/ST will uptake health interventions if they are culturally appropriate and involve marginalized communities in program planning and implementation [22].

There is little doubt that the Government of India will have to make substantial investments in promoting antenatal care and institutional delivery among marginalized communities such as SC/ST. Social and cultural norms are deep-rooted in rural Indian society. It will take much more than improved healthcare access and affirmative action legislation to change hundreds of years of ongoing discrimination and social isolation based on caste [23]. Despite that, this study underscored the need to address what are clearly inequities in the provision maternal health services among these marginalized segments of Indian society.

The study has several limitations. First, data on institutional delivery and antenatal care were self-reported and may have been affected by recall and information bias. Additionally, possession of a BPL card might not be an accurate indicator of socioeconomic status and might be prone to misclassification. Furthermore, since there has been heavy emphasis on promoting institutional birth among rural populations, some respondents may have misreported data on antenatal care and institutional birth because of social desirability bias. Finally, the study was limited in its ability to explore individual reasons for electing antenatal care and institutional delivery. Despite these limitations, the study also has many strengths including a random sample, complete enumeration of all households and high response rate - leading to a more complete sampling of traditionally hard-to-reach groups including SC/ST. Additionally, missing data are unlikely to have altered results due to overall completeness of questionnaires.

## Conclusions

The results of the study suggest that while the Indian Government has made significant progress in increasing antenatal care and institutional deliveries among rural populations, further success in lowering maternal mortality will likely hinge on the success of NRHM programs focused on serving marginalized groups. Health interventions which target SC/ST may also have to address both perceived and actual stigma and discrimination, in addition to providing needed services. Strategies for overcoming these barriers may include sensitization of healthcare workers, targeted health education and outreach, and culturally appropriate community-level interventions. Addressing the needs of these communities will be critical to achieving Millennium Development Goal Five by 2015.

## Abbreviations

ANM: Auxillary nurse midwife; ASHA: Accredited social health activists; BPL: Below poverty level; DLHS: District level household and facility survey; MDG: Millennium development goal; NRHM: National rural health mission; OBC: Other backward caste; PHC: Primary health center; SC/ST: Schedule caste & schedule tribes; TBA: Traditional birth attendants.

## Competing interests

The authors declare that they have no competing interests.

## Authors' contributions

PM and KK were involved in the conception and design of the study. BNK, JF and PA were responsible for acquisition of data. PA and PM analyzed the data. PA, PM, MK and KK drafted the article and all authors (PA, KK, BNK, JF, MK, PM) participated in interpreting the data and critically revising the manuscript for important intellectual content. All authors read and approved the final version of the manuscript to be published. PM has full access to all the data in the study and had final responsibility for the decision to submit for publication.

## Pre-publication history

The pre-publication history for this paper can be accessed here:

http://www.biomedcentral.com/1471-2458/12/30/prepub
